# Understanding low radiation background biology through controlled evolution experiments

**DOI:** 10.1111/eva.12491

**Published:** 2017-06-07

**Authors:** Nathanael Lampe, Vincent Breton, David Sarramia, Télesphore Sime‐Ngando, David G. Biron

**Affiliations:** ^1^ Laboratoire de Physique Corpusculaire CNRS/IN2P3 Université Clermont Auvergne Clermont‐Ferrand France; ^2^ Laboratoire Microorganismes Génome et Environnement UMR CNRS 6023 Université Clermont Auvergne Aubière Cedex France

**Keywords:** adaptation, evolvability, hormesis, low‐dose, radiation

## Abstract

Biological experiments conducted in underground laboratories over the last decade have shown that life can respond to relatively small changes in the radiation background in unconventional ways. Rapid changes in cell growth, indicative of hormetic behaviour and long‐term inheritable changes in antioxidant regulation have been observed in response to changes in the radiation background that should be almost undetectable to cells. Here, we summarize the recent body of underground experiments conducted to date, and outline potential mechanisms (such as cell signalling, DNA repair and antioxidant regulation) that could mediate the response of cells to low radiation backgrounds. We highlight how multigenerational studies drawing on methods well established in studying evolutionary biology are well suited for elucidating these mechanisms, especially given these changes may be mediated by epigenetic pathways. Controlled evolution experiments with model organisms, conducted in underground laboratories, can highlight the short‐ and long‐term differences in how extremely low‐dose radiation environments affect living systems, shining light on the extent to which epimutations caused by the radiation background propagate through the population. Such studies can provide a baseline for understanding the evolutionary responses of microorganisms to ionizing radiation, and provide clues for understanding the higher radiation environments around uranium mines and nuclear disaster zones, as well as those inside nuclear reactors.

## 1 INTRODUCTION

For at least 3.5 billion years, life on Earth has been evolving in ecosystems with differing levels of natural radiation. This forces the question, to what extent is natural radiation, as an abiotic factor, important in the development and evolution of life. Across the planet, the radiation level varies from very low levels in underground spaces (20 nGy/hr), to ambient levels (60–100 nGy/hr), up to the very high levels found in nuclear disaster zones, or where Radon‐rich groundwater leeches to the surface (levels of up to 30 μGy/hr have been recorded in the Ramsar region of Iran, e.g., Ghiassi‐nejad, Mortazavi, Cameron, Niroomand‐rad, & Karam, 2002). The last few decades have provided consistent evidence that the response of living systems to low radiation doses does not follow conventional expectations. Observations of: the bystander effect, where cell signalling can cause otherwise healthy cells to die in response to a radiation dose in a neighbouring cell (Morgan, 2003a, 2003b; Mothersill & Seymour, 2004); genomic instability, where radiation causes an increased rate of genomic changes many generations after irradiation (Limoli, Corcoran, Milligan, Ward, & Morgan, 1999); and transgenerational effects, where hereditary phenotypic changes are observed in the progeny of irradiated cells (Dubrova, 2003), all conflict with an orthodoxy that once asserted that the biological effects of radiation damage can be traced to physical damage of DNA, proteins and cell structures (Feinendegen, Pollycove, & Sondhaus, 2004; Little, Wakeford, Tawn, Bouffler, & Berrington de Gonzalez, 2009; Tubiana, Feinendegen, Yang, & Kaminski, 2006).

Recently, experimentalists have begun observing the behaviour of life in underground laboratories, where the level of radiation exposure can be reduced by five to ten times below the surface level. Such experiments can explore how life has adapted and evolved to the levels of background radiation present on earth today, and reveal how even a slight level of background radiation impacts living systems. Importantly, controlled evolution experiments, revealing how cell populations change with exposure to different radiation environments shed light on the mechanisms responsible for the adaptation of cells to different radiation environments. Typically, one might expect biological experiments conducted in underground laboratories to show no observable differences with experiments conducted at the surface. Conventional measures of radiation risk, such as the Linear‐No Threshold (LNT) model support this, as the natural radiation background is so low that its effects are lost in experimental noise. Nevertheless, biological experiments conducted in underground laboratories have shown that cells cultured across both long and short periods in a low background (LB) environment compared with a standard background (SB) have shown that reducing the radiation background can have detrimental, rather than positive, effects. In long‐duration experiments, a general reduction in the oxidative resistance of cells shielded from environmental background radiation is noticed (e.g., Carbone et al., 2009; Satta et al., 2002), whilst over short durations, a stress response has been observed in cells (Smith, Grof, Navarrette, & Guilmette, 2011; Castillo et al., 2015), which appears with a rapidity that is inconsistent with simple predictions based upon population dynamics and the stochastic nature of radiation damage (Katz, 2016). Thus far, what is missing experimentally is data to link long‐term and short‐term effects of a change in the radiation environment, which quantifies the adaptive and evolutionary mechanisms that can cause these changes.

**Figure 1:**
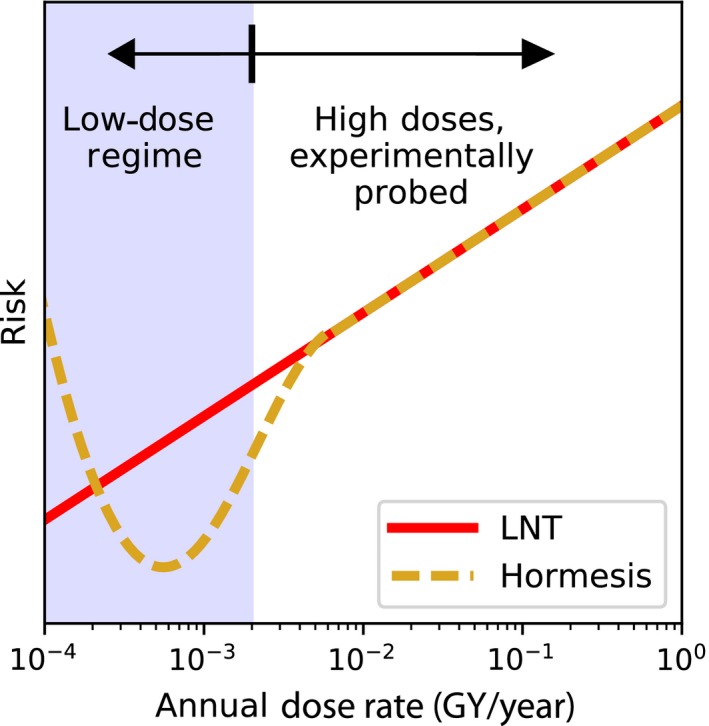
The Linear‐No Threshold model, which describes the risk of cellular damage as linear with increasing dose is experimentally well validated for high doses, but is an extrapolation in the low‐dose regime where biological responses are more difficult to probe. Alternative models, such as radiation hormesis, propose the hypothesis that small radiation doses are stimulatory and often beneficial, reducing the level of risk that would otherwise be present at zero dose

Epigenetics, as a conductor of transgenerational effects is thought to play a significant role in cellular responses at low radiation backgrounds (Merrifield & Kovalchuk, 2013). For example, cells grown at LB have shown higher mutation frequencies than those grown at SB, even six months after being reintroduced to a standard radiation environment (Carbone et al., 2009). This has spurred hypotheses that radiation has a stimulatory effect on cells, activating pathways that defend cells from genetic damage, such as antioxidant production. In this way, radiation behaves as a hormetic agent (Calabrese, 2013; Calabrese & Baldwin, 2003), in direct contradiction to the assumptions of the LNT model (Figure 1).

In and of itself, below background research has its origins in the 1960s (an early review is offered by Luckey, 1982), although modern experiments find their origins in underground research pioneered by Planel et al. (1987) in the French Pyrénées, in caves 200 m below ground operated by the *Centre National de la Recherche Scientifique* at Moulis. Underground, the growth of the protozoan *Peramecium tetraurelia* and the cyanobacteria *Synechococcus lividus* was slowed compared to controls grown at a surface radiation level. Since these early experiments, improved biological methods have become available to experimentalists. These have permitted the quantification of antioxidant activity and gene regulation in cells studied. Developments in related fields have highlighted the extent to which cell communication and epigenetics can impact cells exposed to ionizing radiation, even at low levels. State of the art underground laboratories has begun hosting biological experiments, bringing together these new techniques and paradigms in well controlled and monitored environments. The Gran Sasso National Laboratory in Italy (depth 1,400 m), the Modane Underground Laboratory in France (depth 1,700 m), the Waste Isolation Pilot Plant in the USA (depth 600 m) and SNOLAB in Canada (depth 2,070 m) are involved in active biology projects. In these environments, the level of the radiation background can be dramatically reduced, to be dominated solely by ^40^K present in the biological samples used (Lampe, Marin, et al., 2016).

The aim of this work was to summarize the current state of biological research in underground laboratories, and highlight how an evolutionary approach that quantifies how cells adapt to low and standard radiation environments is essential to advancing the field. We first present an overview of recent low background biological experiments. Next, we discuss the possible mechanisms behind the biological response to low background radiation, attempting to unify the observations made across the many experiments conducted to date. We present some experimental avenues to further elucidate these mechanisms, highlighting the role that multigenerational studies and experimental evolution can play in revealing the ways in which cells change at LB, before highlighting the potential applications of this emerging field of research.

## 2 LOW BACKGROUND BIOLOGY EXPERIMENTS

Low background radiation experiments typically study multiple cell lineages treated in parallel, each originally clones of an ancestral cell type, with equal numbers of cell lines grown at LB and SB. The exact magnitude of the reduction in the radiation background varies between experiments, typically between fivefold and 10‐fold, once contributions to the background from ^40^K in the nutritive medium have been considered. When comparing strains grown in different radiation environments, experimentalists typically target only a few measurements in order to gauge whether the cell populations have responded to the background. As ionizing radiation manifests itself in part as reactive oxygen species‐mediated cellular damage, these tests tend to analyse the presence and activity of proteins implicated in oxidative damage and stress. Mutation induction assays are frequently used, as they allow the response of the cell to oxidative damage to be studied and for similar reasons, the response of cells to radiomimetic toxins is also studied. Finally, cell growth rate is also frequently measured, to see if the low background treatment of the cells has an impact on the performance and viability of the cell as a complete system. Some recent biological experiments conducted in underground and low background conditions have been summarized in Table 1, organized by organism type.

**Table 1:** A selection of recent low background biological experiments, grouped by organism type

Cell Type	Culturing	Experiment	Result
*S. cerevisiae*	120 gen. at LB 120 gen at SB^a^	Mutation challenge from MMS	At high c(MMS), cells cultured in LB have impaired repair
*D. radiodurans*	75 hr at LB 75 hr at SB^b^	Cell growth rate Total Cell protein	Growth was inhibited at LB compared to SB Proteins were reduced at LB
	50 hr at LB 50 hr at SB^c^	Cell growth rate qPCR	Reduced growth at LB compared to SB, which recovered upon re‐introduction to SB Upregulation of heat shock gene dnaK at LB
*S. oneidensis*	50 hr at LB 50 hr at SB^c^	Cell growth rate qPCR	Reduced growth at LB compared to SB, which recovered upon re‐introduction to SB Reaction of stress genes associated with exposure to UV and solar radiation to LB
V79 Chinese Hamster	9 month at LB 9 month at SB^d^	Growth curve Apoptosis following cyclohexamide exposure Antioxidant abundance Mutation induction after γ‐irradiation	Growth rate unchanged between SB and LB. Increased apoptosis compared to control at LB and SB after 3 and 9 month. Significantly increased apoptosis after 3 month at LB compared to SB. Different modulation of antioxidant expression at LB compared to SB No increase in mutation induction at 3 month compared to control, increased mutation induction relative to control and SB at 9 month of LB.
	10 month at LB, then 6 month at SB 16 month at SB^e^	Antioxidant activity Spontaneous mutation frequency	Downregulation of GPx activity in LB and upregulation of GPx activity in SB cells. Increased mutation frequency after 10~mth at LB, increasing further at 16~mth.
Bronchial Epithelial Lung Fibroblast	10 pass. at SB 10 pass. at LB^b^	Protein expression analysis before and after x‐ray exposure	Upregulation of HSP 90B and HSP 70 in LB compared to SB
TK6 Lymphoblastoid	6 month at LB 6 month at SB^f^	Growth curve Micronuclei formation Antioxidant enzyme activity	No dependence on radiation environment More micronuclei formation in LB cells exposed to 2 Gy challenge compared to control. SB cells unchanged compared to control. Reduction in GPx and Catalase enzymes at LB compared to SB, no change in SOD abundance.

a Satta et al. (1995).

b Smith et al. (2011).

c Castillo et al. (2015).

d Satta et al. (2002).

e Fratini et al. (2015).

f Carbone et al. (2009).

Under the hypothesis that removing ionizing radiation from a lineage will reduce the need for ROS scavengers in a cell, attention has been placed upon the superoxide dismutase (SOD) and catalase enzymes, as well as enzymes involved in glutathione regulation — glutathione peroxidase (GPx), glutathione transferase (GST) and glutathione reductase (GSSG‐Rx). Growing V79 Chinese hamster cells in the Gran Sasso underground laboratory, Satta et al. (2002) showed that after 9 months spent culturing independent cell lines in both SB and LB environments, catalase, GPx and GSSG‐Rx were more dominant in the LB culture, whilst SOD levels at LB were expressed at levels between the control and SB levels of expression. Replicating the experiment across a ten month period though, Fratini et al. (2015) found equivalent SOD and catalase levels in lineages from each environment, and significantly reduced levels of GPx. Across such a long‐duration experiment, it is foreseeable that culture ageing could have a stronger effect than the radiation background, explaining this discrepancy. The downregulation of GPx, however, in response to a reduced radiation background is supported by work in human TK6 cells, which have shown over 6‐month growth at LB a significantly decreased quantity of both GPx and catalase enzymes compared to cells grown at SB, whilst the SOD abundance remained constant. In bacterial cells, qPCR analysis of *S. oneidensis* grown over 50 hr at the Waste Isolation Pilot Plant in New Mexico has shown that stress related genes, including those for catalase production, are upregulated by exposure to low radiation environments (Castillo et al., 2015). The same experiments also found that exposure of *Deinococcus radiodurans* to LB upregulated the gene *dnaK*, responsible for producing the heat shock protein HSP70. Intriguingly, the upregulation of HSP70 has also been observed in bronchial epithelial cells and lung fibroblast cells as a result of growth at LB (Smith et al., 2011).

The number of mutants arising in cell populations following irradiation in low background biological experiments can diagnose whether the presence of the natural radiation background is important in the upkeep of biological processes related to DNA repair and prevention of oxidative damage. An increase in the number of mutants with the time spent in the LB treatment relative to the control and SB treatments would indicate that cells removed from the radiation background have lost some of their ability to resist oxidative damage, and suggests that the radiation background has a stimulatory effect on these systems. Irradiating Chinese hamster V79 cells with up to 6 Gy of γ‐radiation from a ^137^Cs source, Satta et al. (2002) measured the number of mutants arising from mutations at the hypoxanthine‐guanine phosphoribosyl transferase (*hprt*) locus, and found that after 3 months of culture at LB and SB, no change in the number of mutants that appeared was apparent compared to a control measurement made at the beginning of the experiment. After 9 months, however, the population grown at LB showed a significant increase in the number of mutants that appeared, including a number of spontaneous mutants that arose even without irradiation. In a similar experiment, Fratini et al. (2015) found a higher rate of spontaneous *hprt* mutations after V79 had been cultured underground for 10 months. Upon reintroduction to a surface‐level radiation environment, where the cells were cultured for another 6 months, the number of spontaneous mutants increased again. This behaviour suggests a long‐term adaptive response to background radiation environments. Additionally, cells that had lost some capacity for repair and oxidative resistance were damaged by the higher oxidative stresses at the surface, to which they did not quickly habituate.

In human TK6 cells, the ability to resist and repair DNA damage was measured by subjecting cells to a 2 Gy dose of X‐rays and measuring the fraction of binucleated cells exhibiting micronuclei following irradiation. Micronuclei formation is indicative of unrepaired or misrepaired chromatin damage. Spontaneous micronuclei formation in populations of cells grown for 6 months at LB and SB, and in a control population from the start of the experiment, shows little variation before irradiation, however, after irradiation micronuclei formation is particularly elevated in the LB population. This further supports the case for a drop in oxidative resistance following culturing of cells for extended time periods in low background environments.

The proportion of aberrant, damaged or apoptotic cells that appear following exposure of cells to toxic agents can often serve as another indication of the ability of cells from a given lineage to recover from DNA damage. One study in the yeast *Saccharomyces cerivisae* showed that cells grown at LB for 120 generations showed a significantly lower ability to resist DNA damage than cells cultured at SB for the same amount of time, when exposed to a high dose of methyl methanosulfonate (MMS), which induces DNA damage by stalling replication forks. In a later study, V79 cells were exposed to cyclohexamide after 3 and 9 months at LB and SB. A significantly increased quantity of hypodiploid cells, indicative of eventual apoptosis, occurred in both LB and SB cells at both time points measured compared to the control sample; however, at 3 months the LB cells were significantly more likely to be hypodiploid than SB cells. Echoing the results of past experiments, this supports the hypothesis that reductions in the ionizing radiation background reduce the resistance of cells to stresses, although here this effect is likely convoluted with a contribution from culture ageing.

Growth curves from cell cultures are an effective way to measure the impact of an environment upon a cell lineage. Planel et al. (1987) found the protozoan *P. tetraurelia* showed a marked increase in its division time when grown at LB compared to SB, whilst a stimulatory effect was observed upon growth when the radiation level was raised beyond the natural by growing cells at higher altitudes where cosmic radiation levels are elevated. This was replicated partially by Kawanishi et al. (2012), who, although unable to replicate inhibited cell growth immediately after cells underwent autogamy, did observe reduced growth rates in *P. tetraurelia* after it had grown at LB for 40 days. Compelling evidence of reduced growth rates in cultures grown at LB has been shown by Castillo et al. (2015) in bacteria, where both *Shewanella oneidensis* and *D. radiodurans* exhibited reduced growth rates within 24 hr of being introduced to LB, compared to a parallel population grown in the same underground laboratory with a simulated SB environment. Additionally, the LB populations had lower maximum optical densities at the end of the exponential growth phase. It was also demonstrated in this work that the higher growth rate at SB could be rapidly recovered by transferring the population grown at LB back to the SB environment. Studies of growth rates in mammalian cells have not, however, indicated a clear difference in growth rate between cells grown at LB and SB. Neither TK6 cells (Carbone et al., 2009) nor V79 cells (Satta et al., 2002) showed a significant difference in doubling time after being cultured over months at LB and SB compared to the doubling time measured at the start of the experiment.A continual concern in these experiments is that an external factor causes the change in observed behaviour, rather than the radiation dose. Between two different laboratories, pressure and humidity differences may induce a cellular response if they are not well controlled, and careful control of lighting is necessary to ensure that the cells in each environment do not adopt different circadian rhythms (Bell‐Pederson et al., 2005). The recent work of Castillo et al. (2015) is particularly noteworthy as it shows the effects of the radiation background, whilst growing two cultures in environments as identical as possible, apart from the radiation background, by introducing a source into an underground environment.

## 3 MECHANISMS FOR LOW BACKGROUND STRESS RESPONSES

Repeated observations of changing biological outcomes from different treatments indicate that organisms are in some capacity able to sense and respond to changes in their radiation environment even at very low doses. Largely, experimental work has been focused on identifying the population level outcomes of these changes in radiation background. These responses often align with the theory that radiation has a hormetic effect upon cells, being a stimulant at very low doses and toxic at large doses. The mechanisms that give rise to such observables in response to the radiation environment are not well known. Part of the difficulty in identifying the mechanisms responsible for the low background stress response is the possible epigenetic origin for these effects. Such a hypothesis would find support in the already explored epigenetic origins of other low‐dose radiation responses such as the bystander effect (Morgan, 2003a, 2003b) and well‐documented transgenerational effects of radiation exposure (Dubrova, 2003).

The study of both prokaryotic and eukaryotic cells in low background environments allows better identification of the enzymatic responses to low background radiation, by allowing similar responses to be compared across domains. In particular, across a variety of cell types, regulatory changes in the abundance of H_2_O_2_^−^ related antioxidants are commonly seen, whilst the abundance of SOD, used in the reduction of O_2_^·−^ rarely changes. This itself is likely explained by the initial distribution of reactive oxygen species following water radiolysis, which favours H_2_O_2_ relative to the production of O_2_^·−^ (Ferradini & Jay‐Gerin, 1999; Ward, 1988). This gives one physical motivation for the decreased expression of GPx and catalase observed in TK6 and V79 cells. In *S. oneidensis*, where catalase activity is overexpressed according to qPCR after 24 hr at LB, this could be consistent with a reduction in catalyse transcription as its rate of consumption is reduced, although this hypothesis is not consistent with the infrequency with which radiation interacts with bacterial cells over a short‐time period. Ultimately, the mechanism for this is likely tied to a change in cellular regulation, rather than consumption of antioxidants by radicals, as over long time periods, many‐fold changes in the radiation background induce only minimal changes in ROS abundance (Smith, Willey, & Hancock, 2012).

Whilst radiation dose is typically envisaged as a continuous deposit of energy in a volume, in low‐dose regimes and at cellular levels, energy depositions in cells are best described by a stochastic model. This was underlined by Katz (2016) and has been further explored in the context of underground laboratories by Lampe, Biron, et al. (2016). These investigations show that radiation tracks from background sources on the order of 100 nGy/hr interact rarely with bacterial cells, affecting less than 0.1% of the population per day. Even in larger animal cells, the background strikes only a tiny fraction of cells daily. The emergence of population level responses to a reduction in the radiation level is difficult to explain based on the individual alone, unless some form of communication occurs between cells to permit the responses observed. Observations of a bystander effect in mammalian cells have shown that there is precedent for tissues to react as a whole when subjected to localized irradiation (e.g., Baskar, 2010), and Castillo et al. (2016) advance that a similar mechanism, possibly driven by bacterial cell‐to‐cell communication methods could be responsible for population level responses in bacteria. Hypothetically, cell signalling could magnify this effect, although further research would be needed to assert this. Meanwhile, the emergence of a low growth rate in bacteria rather than in more complex cells could be related to the differing reproductive mechanisms of eukaryotic and prokaryotic cells. This may also be relevant for the protozoa *P. tetraurelia* if its retarded growth at LB is related to slower growth of its bacterial food source.

A rapidly growing body of epigenetic studies continually indicates that environment can have a profound impact upon cells in ways that are subtle and oft‐times hard to detect (e.g., Jirtle & Skinner, 2007; Merrifield & Kovalchuk, 2013). Given the collected evidence for a biological response to the background, it is reasonable to suggest that epigenetic responses to ionizing radiation play a significant role in low background biological experiments. This is concordant firstly with the low frequency with which ionizing radiation strikes cells. As the entire population responds to the background, it is possible that a response to being impacted by ionizing radiation is inherited epigenetically across multiple generations. Given the potentially violent impact of ionizing radiation upon an individual cell, it is possible to imagine a scenario where natural selection favours the development of a strong defensive response to irradiation that lasts across multiple generations. In particular, changes by a factor of two to ten in the radiation background may still leave the chance of being impacted by radiation in a given cells lifetime quite small (≤5%), so in order to be sensitive to this particular effect, a strong intergenerational memory of radiation is required. Communication between cells would increase the ability of cells to detect their radiation environment and respond to it. This has been observed in the bystander effect and may even be regulated by similar mechanisms. Intercellular gap junctions have been shown to mediate information related to radiation exposure between cells (Shao et al., 2003), while within a medium, the abundance of ROS (Azzam, De Toledo, & Little, 2003; Mothersill & Seymour, 2004), Ca^2+^ ions (Lyng, Maguire, McClean, Seymour, & Mothersill, 2006) and short RNA sequences (Ilnytskyy & Kovalchuk, 2011) have all been implicated in transmission of radiation‐related information.

An observation largely missing from our discussion thus far is the amount of reactive oxidative species generated by background radiation. Recent work has shown that background ionizing radiation, even in nuclear disaster zones, does not consume a sufficient amount of antioxidants to account for long‐term decreases in antioxidant concentration seen in environmental studies (Smith et al., 2012). When organisms and radiation together are pictured as a bulk material, radio‐induced chemical species cannot account for the reductions in antioxidant abundancies observed, for example, in barn swallows (Møller, Surai, & Mousseau, 2005). The variations in antioxidant abundance in response to different radiation backgrounds may require more detail than a continuum model offers to be understood (this has been explored in part by Lampe, Biron, et al., 2016). At the level of a cell, radiation‐induced radicals are created stochastically, whenever radiation traverses the cell. Radiation dose defines the frequency with which sometimes large quantities of radicals are created in cells. Changes in the dose corresponding to the rate at which violent radical creation occurs within cell may in fact be what encourage cells to change their internal antioxidant regulation.

There remains a significant amount of work to be done in understanding the mechanisms behind the low radiation background response of cells. While a decreased need for antioxidants as the quantity of radiation‐induced oxygen radicals decreases could drive an adaptive response that leads to lower antioxidant production (though the decreased metabolic cost of this would be thought to spur an increase in growth rate, the opposite of what has been observed in bacteria, this image is in conflict with the activation of other stress sites following exposure to LB, the statistical infrequency with which radiation hits cells and the relatively low ROS yields of background radiation. Further work needs to be done to explore how a population‐wide response emerges in a given lineage from a phenomenon that has its origins in relatively rare individual effects. Equally, a clear picture has yet to emerge of which antioxidants consistently change in their regulation following radiation deprivation. Catalase and GPx are strong candidates but are not universally shown to change compared to an SB control. Conversely, measurements of the resistance of a cell population to DNA damage have shown a clear tendency towards lower damage tolerance in cells grown at LB. While this is a cruder biological end‐point than gene expression, protein abundance and other measures of cell regulatory systems, its consistency instils confidence that cell populations can sense their radiation environment.

## 4 PERSPECTIVES FOR FUTURE EXPERIMENTS

The capacity for cell populations to respond to their radiation environment across both short‐time and long time durations warrants further investigation for several reasons. Importantly, future space exploration is likely to expose humans and other biota to a wide variety of radiation environments. Technologically, the potentially high sensitivity of biological systems to the radiation background as demonstrated in bacteria could lead to high accuracy organic dosimeters. New developments in either of these domains require a strong mechanistic understanding of what is causing biological systems to respond to low background radiation. Experiments using qPCR to quantify gene expression in parallel with protein density offer a promising first glance at what is underpinning changes in cell populations grown underground. An important missing link mechanistically is uncovering how changes in already infrequent interactions between radiation and cells can rapidly change the dynamics of a whole lineage. Part of this can be solved with more frequent time sampling. Bacterial cells have been studied across time windows of up to a few days, whilst mammalian cells have largely been studied at intervals ranging from 3 to 10 months. It is difficult then to judge whether effects are adaptive, or passed on epigenetically in response to the environment, or whether changes in cells at grown at LB are merely a stress response that persists until cells are returned to SB. Thus far, experiments have indicated that both of these hypotheses are possible. Documenting in detail the transition of a cell from a SB to a LB state and back again could reveal some of the mechanisms behind the changes experimentalists have observed.

Experiments showing LB behaviour persisting 3 months after cells grown at LB are returned to SB tends to favour the conclusion that there is a level of heritability to the radiation background response. The observation of such a response merits further investigation into its origin. Genome sequencing of both the ancestor cell line and its developed daughter could indicate whether this change has its origins in the genome or in its interpretation. The identification of behavioural markers of epigenetic trends in the radiation response could be incredibly insightful in understanding the mechanism underlying these responses. Experimental controls that clearly identify heritable and nonheritable changes in population dynamics can aid in determining to what extent the changes seen at LB are direct responses to an environment, and to what extent they are driven by selection or epigenetic mechanisms.

A challenge for this field is also the identification of a suitable control species that is robust to reductions in the radiation environment. While other environmental parameters in experiments can be monitored to ensure they remain constant between radiation environments, such as air pressure, temperature and culture conditions, the consistency of the environment could be assured by having a cell line that does not respond to variations in the radiation environment. This may, however, be a difficult endeavour as already *D. radiodurans*, which is amongst the most radio‐resistant bacteria known (Battista, 1997), has been shown to be affected by changes in radiation environment. Eukaryotic cells, being more developed than bacteria, may offer a better candidate for a control species; however, there remains significant work to be done in identifying which cells respond least to radiation. Alternatively, control mechanisms in cells may be identifiable. The significantly increased yield of H_2_O_2_ following water radiolysis compared to O_2_^·−^ may allow studies of antioxidant behaviour to use SOD abundance as a control for nonradiative oxidative stress, while measuring changes in GPx abundance in response to radiation.

Understanding the role of ROS and antioxidant regulation in environmental radiobiology remains a challenge for the field. Changes in antioxidant abundancies are linked to more than just an accumulation or depletion of proteins caused by changes in radical induction. To better understand the cellular ROS response to ionizing radiation, microbeams may be used to conduct single cell studies. Although it may be difficult to calculate ROS yields directly, the behaviour of antioxidants in singly irradiated cells both immediately after irradiation, and many days after irradiation could provide clues for how radiation events impact antioxidant dynamics.

Scope also exists to precisely control the radiation level cells experience at LB to identify when LB behaviours begin to manifest. Currently, most experiments to date are conducted at a level determined by the nutritive medium cells are grown in. After suppression of external radiation, trace amounts of ^40^K in biological media expose cells to β^−^‐rays. The response of cells to radiation levels between this level and SB could indicate whether the transition to a stressed cell state at LB is a binary switching effect, or whether it happens gradually, both of which could shine light upon the underlying biological mechanism by which populations respond to their radiation environment. Beyond this, studies using nutritive media containing ^39^K only could be conducted to see if removal of even greater levels of radiation accentuates cellular responses to radiation suppression. A study by Gevertz, Friedman, Katz, and Kubitschek (1985) using nutritive media alternatively enriched and lacking in ^40^K found that the isotope's presence had no impact on the mutation rate of *Escherichia coli;* however, the presence of a surface‐level radioactive background likely reduced the significance of the reduction in ^40^K.

Given the unexpected results that occur in biological systems in the absence of radiation, it is also worthwhile to consider whether conducting long‐term evolution experiments in underground laboratories can reveal if the evolutionary behaviour of a cellular system is impacted by the radiation environment. Steinhauser (2015) suggests that, due to the randomness with which radiation damages cells at the background levels present on earth, the evolutionary response to radiation must occur at an individual, cellular level. In addition to individual cellular responses to radiation, experiments observe coordinated responses to ionizing radiation in large cell populations. Moreover, given radiation's capacity to damage DNA in a reasonably random way, it is possible that high radiation levels could change the mutation rates of species, the stresses to which species adapt and the landscape of potential mutations available to an organism. At the high radiation limit, it has been shown that when radiation is a strong selective pressure, *E. coli* can evolve extreme radio‐resistance (Byrne et al., 2014). At less extreme levels, it is possible, however, that radiation could have similar effects to mutator genes in changing the long‐term evolutionary behaviour of a species (de Visser et al., 1999).

Controlled long‐term experimental evolution experiments offer an excellent tool to study many of the questions raised by LB biology experiments. By offering an experimental model that is reproducible across independent lineages, the origins of changes, genetic or otherwise, that appear in cell populations can be thoroughly scrutinized. Many model organisms exist for which long‐term controlled evolution data and protocols are already available, such as the prokaryote *E. coli* (Barrick et al., 2009; Lenski, Rose, Simpson, & Tadler, 1991), the eukaryote *S. cerivasae* (Ferea, Botstein, Brown, & Rosenzweig, 1999; Sonderegger & Sauer, 2003) and even the fruit fly *Drosophila melanogaster* (Burke et al., 2010). Advancing along this path, experiments are currently underway in the Modane Underground Laboratory to reproduce *E. coli* based long‐term evolution experiments in different radiation environments (Lampe, Biron, et al., 2016). Through these pilot experiments, we hope to clarify the extent to which LB environments change the evolutionary comportment of a simple bacterial population. In particular, it is important to establish whether conducting experiments at LB affects the mutation rate by reducing the interactions between cells and ionizing radiation. While at normal backgrounds the radiation‐induced mutation rate typically contributes to only a fraction of the total mutation rate, such an experiment will show whether radiation makes certain patterns of mutation more likely than others, and will be able to establish the viability of long‐term evolution experiments as a tool for understanding the broad array of cellular responses to low radiation backgrounds.

## 5 POSSIBLE APPLICATIONS OF LOW BACKGROUND STUDIES

Studying life in the near absence of radiation allows the suppression of an abiotic factor to which life has adapted over millenia. When considering the impact of ionizing radiation on environmental systems, it can be difficult to separate the role of ionizing radiation as a selective pressure (and thus a target of natural selection), and a mutagenic agent. The confusion of these two aspects of ionizing radiation is notably a source of concern for the public in relation to nuclear power. The extent to which these two factors interact can be explored by removing or fixing the background and, for example, introducing mutator genes to change the mutation rate in a radiation‐agnostic manner (de Visser et al., 1999). The relevance of this question is highlighted in studies from environmental toxicology, where a picture is emerging that microbial responses to the radiation background are linked both to exposure time and the dose rate (Siasou, Johnson, & Willey, 2017). Here again, clear measurements of radiation responses to very low radiation levels can provide clarity as to how the impacts of natural radiation should be quantified.

Beyond the applicability of this work to understanding radiation's abiotic role in adaptation and evolution, it's tempting to consider the applicability of underground laboratories towards preserving DNA. For most organisms, the lower limit of the mutation rate is constant (Drake, 1991; Drake, Charlesworth, Charlesworth, & Crow, 1998), with thermophiles showing a mutation rate about ten times lower than this, likely because a higher fraction of mutations is harmful at high temperatures (Drake, 2009). A large part of why the mutation rate does not become smaller than these limits is because genetic drift, and to a lesser extent, radiation damage, provide a threshold level of genetic variation and mutations in the population that it is too resource intensive to overcome, relative to the benefits a more perfect duplication rate would give. In underground laboratories, one source of mutational “noise” can be eliminated, allowing us to speculate whether very low radiation environments could be part of an experimental scenario that encourages the emergence of perfect or near perfect genome duplication.

## 6 CONCLUSION

Low background biological research has consistently shown that despite the natural radiation background already being incredibly small, it is nevertheless significant enough for living systems to sense it and respond to it. The adaptive and hormetic effects that are noticed at low background are not yet well understood, and significant experimental work is needed to better clarify them. Experimental approaches that draw from evolutionary biology are well adapted to this task, as epigenetic and genetic changes may occur at low radiation backgrounds. Whole sequence genotyping, proteomics, multigenerational studies and long‐term evolution experiments are some of the mechanisms that may be applied in approaching this problem.
